# Апробация и валидация русской версии опросника для оценки качества жизни у больных первичным гиперпаратиреозом — PHPQoL

**DOI:** 10.14341/probl12714

**Published:** 2021-01-19

**Authors:** И. Н. Гладкова, В. Ф. Русаков, Р. А. Черников, Ю. В. Карелина, Т. П. Никитина, С. М. Ефремов, Т. И. Ионова

**Affiliations:** Санкт-Петербургский государственный университет, Клиника высоких медицинских технологий им. Н. И. Пирогова; Санкт-Петербургский государственный университет, Клиника высоких медицинских технологий им. Н. И. Пирогова; Санкт-Петербургский государственный университет, Клиника высоких медицинских технологий им. Н. И. Пирогова; Санкт-Петербургский государственный университет, Клиника высоких медицинских технологий им. Н. И. Пирогова; Санкт-Петербургский государственный университет, Клиника высоких медицинских технологий им. Н. И. Пирогова; Санкт-Петербургский государственный университет, Клиника высоких медицинских технологий им. Н. И. Пирогова; Санкт-Петербургский государственный университет, Клиника высоких медицинских технологий им. Н. И. Пирогова

**Keywords:** первичный гиперпаратиреоз, качество жизни, опросник PHPQoL, русская версия

## Abstract

**Обоснование:**

Обоснование. Оценка качества жизни до и после хирургического лечения при первичном гиперпаратиреозе (ПГПТ) может быть использована для комплексной оценки эффекта терапии, а также мониторинга состояния больного после операции, в том числе в реальной клинической практике.

**Цель:**

Цель. Целью исследования являлась валидация русской версии опросника PHPQoL для оценки качества жизни у больных первичным гиперпаратиреозом (ПГПТ) и его апробация в отечественной популяции больных ПГПТ с целью дальнейшего применения в клинической практике и научных исследованиях.

**Материалы и методы:**

Материалы и методы. В соответствии с международными стандартами проведены процедура языковой и культурной адаптации опросника PHPQoL, а также тестирование в фокусной группе больных ПГПТ с последующей оценкой психометрических свойств инструмента — надежности, валидности и чувствительности.

**Результаты:**

Результаты. В исследовании участвовали 65 больных ПГПТ (средний возраст — 52,3±10,5 года, 97% — женщины), из которых у 67,7% установлена манифестная форма заболевания, у 35,4% пациентов имелась умеренная или тяжелая форма гиперкальциемии. Все пациенты заполняли валидированную нами русскую версию опросника PHPQoL до паратиреоидэктомии. Треть пациентов заполнили опросник повторно через 3 мес после операции. В ходе апробации опросника продемонстрированы высокие показатели его внешней и содержательной валидности, а также показана устойчивая структура инструмента, свидетельствующая об удовлетворительной конструктной валидности опросника. Также показана способность опросника определять различия показателей симптомов/проблем ПГПТ между пациентами в зависимости от выраженности клинических признаков заболевания и в процессе лечения. Продемонстрирован положительный эффект хирургического лечения на качество жизни больных ПГПТ после операции.

**Заключение:**

Заключение. Результаты, полученные в ходе исследования, свидетельствуют о надежности, валидности и чувствительности русской версии опросника PHPQoL и приемлемости его дальнейшего использования в научных исследованиях и клинической практике в отечественной эндокринологии.

## Abstract (en)

**Background:**

Background: Quality of life (QoL) assessment before and after surgical treatment in patients with primary hyperparathyroidism (PHTP) may be useful for comprehensive evaluation of the treatment effect, as well as for monitoring of the patient’ condition after surgery, including in real clinical practice.

**Aim:**

Aim: The aim of the study was to validate and test the Russian version of the PHPQoL questionnaire for assessment of the quality of life (QoL) in patients with primary hyperparathyroidism (PHPT) for further application in clinical practice and research in Russia.

**Materials and methods:**

Materials and methods: The linguistic and cultural adaptation of the PHPQoL questionnaire was carried out in accordance with international guidelines. Psychometric properties of the tool, namely, its reliability, validity and sensitivity were tested in the focus group of patients with PHPT

**Results:**

Results: In the whole, 65 patients with PHPT were involved into the study (mean age — 52.3 ± 10.5 years, 97% — ­women): 67.7% patients were symptomatic, 35.4% patients had moderate or severe hypercalcemia. All the patients filled out the Russian version of PHPQoL before parathyroidectomy. One third of patients filled out the Russian version of PHPQoL twice — before surgery and 3 months after surgery. Satisfactory external and content validity of the Russian version of PHPQoL was demonstrated. Its stable structure confirmed satisfactory construct validity of the questionnaire. The ability of the tool to determine differences in severity of symptoms/problems due to PHPT before and after treatment was revealed. The positive effect of the surgery on QoL in PHPT patients was shown.

**Conclusion:**

Conclusion: The results obtained during the study confirm that the Russian version of PHPQoL is a reliable, valid and sensitive tool. Feasibility and applicability of its use in research and clinical practice in Russian endocrinology settings has been demonstrated.

## ОБОСНОВАНИЕ

Первичный гиперпаратиреоз (ПГПТ) — эндокринное заболевание, которое характеризуется избыточной секрецией паратиреоидного гормона (ПТГ) при верхне-нормальном или повышенном уровне кальция крови вследствие первичной патологии одной или нескольких околощитовидных желез (ОЩЖ) [1]. В Российской Федерации отсутствует единый регистр заболеваемости ПГПТ, однако проведенные в странах Западной Европы и Северной Америки эпидемиологические исследования показали, что ПГПТ занимает по частоте 3-е место после сахарного диабета и патологии щитовидной железы. Распространенность ПГПТ среди взрослого населения в популяции около 1%, чаще болеют женщины старше 55 лет — до 2% [2]. Манифестная форма заболевания характеризуется полиорганностью поражения. Для манифестной формы ПГПТ типичны патологические изменения в костях — с развитием гиперпаратиреоидной остеодистрофии, почках — в виде нефролитиаза и нефрокальциноза. Также наблюдаются изменения со стороны желудочно-кишечного тракта с развитием язвенной болезни желудка и двенадцатиперстной кишки. Для пациентов со всеми формами ПГПТ характерна более высокая сердечно-сосудистая смертность вследствие прогрессирования атеросклероза и артериальной гипертензии.

Гораздо меньше известны и требуют дальнейшего изучения вопросы о распространенности и характере патологических изменений со стороны нервно-психической сферы. К ним относятся интеллектуально-мнестические расстройства, депрессия, неустойчивость настроения и др. [1][3–5].

Наряду с манифестной, значительно чаще встречается мягкая (малосимптомная, бессимптомная) форма ПГПТ, при которой отсутствуют характерные для манифестной формы проявления со стороны костей, почек и внутренних органов. Несмотря на то что некоторые пациенты с мягкой формой ПГПТ рассматриваются как «бессимптомные», у них, тем не менее, могут иметь место различные проблемы, связанные с заболеваниями, которые способствуют ухудшению качества жизни больных и которые целесообразно оценивать и мониторировать [6].

Хирургическое лечение является радикальным и наиболее эффективным методом лечения ПГПТ [7][8]. По данным ряда исследований показано, что паратиреоидэктомия (ПТЭ) приводит к улучшению показателей качества жизни у больных ПГПТ [9–13]. Использование информации, полученной напрямую от пациента, при анализе клинических проявлений заболевания и оценке динамики симптомов в процессе лечения способствует реализации пациентоориентированного подхода при ведении больных ПГПТ. Оценка качества жизни до и после хирургического лечения при ПГПТ может быть использована для комплексной оценки эффекта терапии, а также мониторинга состояния больного после операции, в том числе, в реальной клинической практике.

В этой связи представляется актуальным мониторинг качества жизни и симптомов у больных ПГПТ с использованием стандартизированных опросников. Одним из стандартизированных опросников для оценки качества жизни у больных ПГПТ, в том числе до и после хирургического лечения, является опросник для оценки качества жизни при гиперпаратиреозе — PHPQoL [14]. Опросник содержит 16 вопросов, 9 из которых касаются физического функционирования больного, а 7 — эмоционального функционирования. Варианты ответов представляют собой баллы по шкале Ликерта для оценки того, как часто беспокоила пациента та или иная проблема в течение последнего месяца (0 баллов — проблема беспокоила все время, 4 балла — проблемы никогда не было). Сумму баллов по шкалам Ликерта для 16 вопросов преобразуют с помощью процедуры стандартизации в суммарный показатель качества жизни, значения которого могут варьировать от 0 до 100 — чем выше суммарный балл, тем лучше качество жизни. Таким же способом рассчитывается физический компонент качества жизни (стандартизированная сумма баллов по первым 9 вопросам опросника) и психический компонент качества жизни (стандартизированная сумма баллов по остальным 7 вопросам опросника).

Согласно данным проведенных исследований, опросник PHPQoL является надежным, валидным и чувствительным инструментом для оценки симптомов и проблем у больных ПГПТ, в том числе для мониторинга состояния пациентов после операции и оценки эффекта хирургического лечения, при разной степени клинических проявлений заболевания [15][16]. Так, в работе Webb S.M. и соавт. с помощью опросника PHPQoL изучали качество жизни пациентов с ПГПТ [15]. Всего в исследование включено 183 больных. Группу А (n=104, средний возраст — 60 лет, мужчины/женщины — 23/81, средняя длительность времени от момента постановки диагноза до операции — 1,7 года) составили пациенты, которым проведено медикаментозное или оперативное лечение; группу Б (n=78, средний возраст — 63 года, мужчины/женщины — 14/64, средняя длительность времени от момента постановки диагноза до операции — 1,7 года) — пациенты, находящиеся в стабильном состоянии и не требующие лечения. В результате исследования продемонстрировано, что показатели качества жизни пациентов с ПГПТ после начала медикаментозного лечения или после операции улучшаются, согласно данным опросника PHPQoL, уже через 3 мес, и положительные изменения сохраняются через 6 и 12 мес [15]. В недавно проведенном исследовании Somuncu E. и соавт., в котором приняли участие 50 пациентов с ПГПТ (средний возраст — 60 лет, мужчины/женщины — 8/33, средняя длительность времени от момента постановки диагноза до операции — 11 мес) с помощью опросников PHPQoL и SF-36 показано значимое улучшение качества жизни пациентов через 12 мес после ПТЭ [16]. В настоящее время, по данным литературных источников, имеются версии опросника PHPQoL на английском и датском языках. С учетом большого практического значения применения данного опросника в научных исследованиях и клинической практике при ведении пациентов с ПГПТ представляется своевременной валидация русской версии PHPQoL.

Разработка опросника на другом языке состоит из двух этапов. На первом этапе проводится языковая и культурная адаптация, на втором — оценка психометрических свойств (валидация) созданного опросника. Языковая и культурная адаптация опросников — это многоступенчатый процесс создания эквивалентного оригиналу инструмента на русском языке с учетом этнолингвистических особенностей популяции. Данный процесс выполняется не только специалистами, но предполагает также участие пациентов. Качество проведения данного этапа определяет в дальнейшем психометрические свойства инструмента [17]. После проведения этих двух этапов опросник PHPQoL может быть рекомендован для применения в научных исследованиях и клинической практике у больных ПГПТ в России.

## ЦЕЛЬ ИССЛЕДОВАНИЯ

Цель данного исследования — языковая и культурная адаптация и валидация русской версии опросника PHPQoL и его апробация в популяции больных ПГПТ с целью его дальнейшего применения в клинической практике и научных исследованиях.

## МАТЕРИАЛЫ И МЕТОДЫ

## Место и время проведения исследования

Исследование проводили в период с сентября 2019 г. по октябрь 2020 г. на базе отделения эндокринной хирургии Клиники высоких медицинских технологий им. Н.И.  Пирогова СПбГУ.

## Методы

На первом этапе исследования провели процедуру языковой и культурной адаптации русской версии опросника PHPQoL после получения разрешения от автора опросника S.M. Webb (Отдел эндокринологии госпиталя Сант Пау, Барселона). Для перевода и адаптации использовали англоязычную версию опросника PHPQoL. Перевод, языковую и культурную адаптацию опросника PHPQoL проводили в соответствии с современными международными рекомендациями. Этапы создания русской версии опросника PHPQoL представлены на рисунке 1.

**Figure:**
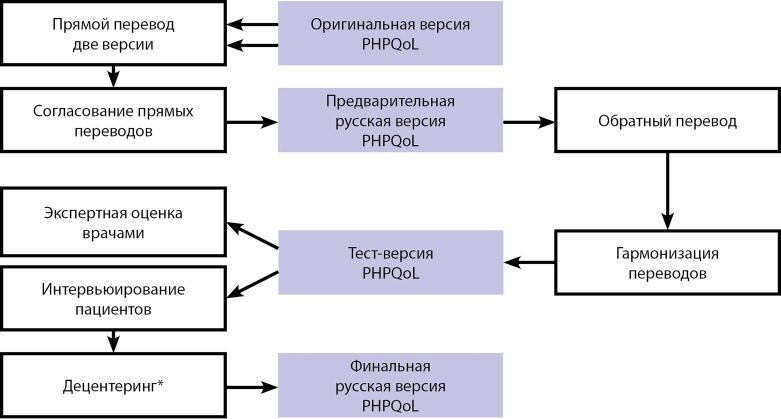
Рисунок 1. Этапы языковой и культурной адаптации опросника PHPQoL в рамках создания русской версии инструмента.*Примечание: децентеринг — это внесение изменений в опросник по результатам интервьюирования.

Процедуру создания русской версии опросника PHPQoL осуществляли таким образом, чтобы обеспечить эквивалентность финальной версии опросника оригиналу и адаптировать опросник к этнолингвистическим особенностям популяции. Результаты перевода и адаптации позволили получить русскую версию опросника, которая соответствовала оригиналу по следующим параметрам: функциональная эквивалентность, структурная эквивалентность, операционная эквивалентность.

Тест-версию PHPQoL проверяли в фокусной популяции пациентов с ПГПТ в процессе индивидуального интервьюирования. В зарубежных рекомендациях этот этап называют когнитивным дебрифингом [17]. Данный этап состоит в тестировании опросника в рамках создания его русской версии на основании мнения пациентов. Цель тестирования (когнитивного дебрифинга) русской версии PHPQoL — максимально приблизить концепцию инструмента к культурным и языковым традициям и особенностям популяции больных ПГПТ в России. Интервьюирование (тестирование) больных проводили на базе отделения эндокринной хирургии Клиники высоких медицинских технологий им. Н.И. Пирогова СПбГУ после подписания пациентами информированного согласия. Ключевыми аспектами при тестировании PHPQoL в процессе интервьюирования пациентов по каждому пункту опросника являлись:

В рамках этого этапа также проверяли внешнюю валидность русской версии опросника PHPQoL — на основании мнения пациентов, а также содержательную валидность — на основании мнения специалистов-эндокринологов. Показатели внешней валидности оценивали по отдельным категориям: понятности, легкости и удобству и полноте оценки, а также по общему (среднему) показателю валидности. При оценке содержательной валидности определяли понятность, легкость и удобство оценки, а также общую информативность опросника на основании мнения специалистов.

На втором этапе работы провели апробацию русской версии опросника PHPQoL в группе больных ПГПТ, которым показано хирургическое лечение с проведением ПТЭ с оценкой психометрических свойств инструмента. В рамках апробации вновь анализировали понятность и легкость заполнения русской версии опросника PHPQoL пациентами, оценивали качество данных при заполнении опросника, а также определяли суммарный балл качества жизни по опроснику в фокусной популяции больных ПГПТ.

В исследование включали пациентов 18 лет и старше с подтвержденным диагнозом ПГПТ, при наличии показаний для проведения хирургического лечения, а также при условии согласия на участие в исследовании и способности пациентов заполнить опросники. Перед началом исследования было получено письменное информированное согласие на участие в исследовании от каждого пациента. Критериями исключения были следующие — наличие серьезных сопутствующих заболеваний, симптомы которых, по мнению врача, преобладали над симптомами основного заболевания (при декомпенсации хронических заболеваний), а также наличие у пациентов когнитивных нарушений, препятствующих заполнению опросников.

Пациенты заполняли опросник PHPQoL, а также общий опросник оценки качества жизни RAND SF-36 при поступлении на отделение эндокринной хирургии до операции. Для оценки чувствительности опросника к изменению во времени некоторые больные заполняли опросник PHPQoL повторно через 3 мес после ПТЭ. Врачи вносили социодемографическую и клиническую информацию в карту больного.

## Методы оценки психометрических свойств опросника

Валидация русской версии опросника PHPQoL включала оценку его психометрических свойств — валидности, надежности и чувствительности. Анализ валидности включал оценку конструктной, дискриминантной, критериальной и конвергентной валидности. Конструктную валидность опросника PHPQoL проверяли с помощью изучения его структуры факторным анализом. Анализ надежности проводили с помощью оценки внутреннего постоянства путем вычисления коэффициента α-Кронбаха (коэффициента внутренней согласованности). Дискриминантную валидность оценивали методом известных групп (known-groups method). Для этого сравнивали суммарный балл по опроснику PHPQoL у пациентов с бессимптомной и манифестной формами заболевания. Для оценки критериальной валидности проводили анализ корреляций между суммарным баллом по PHPQoL и количеством испытываемых пациентами симптомов. Конвергентную валидность оценивали с помощью корреляционного анализа между суммарным баллом PHPQoL и шкалами опросника RAND SF-36, который рассматривали как золотой стандарт для оценки качества жизни у пациентов с хроническими заболеваниями. Опросник SF-36 состоит из 36 вопросов. В результате шкалирования формируются 8 шкал: физическое функционирование, ролевое физическое функционирование, боль, общее здоровье, жизнеспособность, социальное функционирование, ролевое эмоциональное функционирование и психическое здоровье. Показатели по шкалам SF-36 выражают в баллах от 0 до 100; чем выше балл по шкале опросника SF-36, тем лучше показатель качества жизни. Для оценки чувствительности PHPQoL проводили сравнение изменений суммарного показателя по опроснику PHPQoL через 3 мес после операции по сравнению с его значением до операции.

## Статистический анализ

Данные представлены в виде количества наблюдений, среднего арифметического значения, стандартного отклонения, диапазона и процентных долей. При выборе критерия проверки статистической значимости различий между анализируемыми показателями основывались на характере распределения данных. Проверка нормальности распределения исследуемых выборок проводилась с использованием критерия Шапиро–Уилка. При сравнении двух групп пользовались критерием сравнения для двух несвязанных выборок — t-критерием Стьюдента. При сравнении показателей в динамике использовали t-критерий Стьюдента для двух связанных выборок. Для анализа структуры опросника PHPQoL проведен разведочный факторный анализ с использованием метода главных компонент и способа вращения варимакс при условии минимального собственного значения фактора ≥1. Для анализа использовали все опросники PHPQoL, в том числе заполненные пациентами в двух точках исследования. Для оценки связи между показателями использовали ранговую корреляцию Спирмена. Корреляционную связь рассматривали как очень слабую при r=0–0,3, слабую — при r=0,3–0,5, среднюю — при r=0,5–0,7, высокую — при r=0,7–0,9, очень высокую — при r=0,9–1. Все тесты двусторонние, различия между сравниваемыми группами признавали статистически значимыми при уровне p<0,05. Статистический анализ проведен с использованием программного обеспечения SPSS 23.0 и Statistica 10.0.

## Этическая экспертиза

Исследование одобрено Комитетом по биомедицинской этике Клиники высоких медицинских технологий им. Н.И. Пирогова СПбГУ (выписка из протокола №08/19 от 15.08.2019).

## РЕЗУЛЬТАТЫ

## Языковая и культурная адаптация опросника PHPQoL

Прямой перевод инструмента PHPQoL был выполнен независимо двумя переводчиками, носителями русского языка, которые имели опыт перевода специальной медицинской литературы. В результате были разработаны два варианта прямого перевода PHPQoL. При создании предварительной тест-версии опросника были выбраны оптимальные формулировки, которые были предложены переводчиками с учетом культурных и этнолингвистических особенностей популяции. Далее предварительная тест-версия PHPQoL была утверждена в ходе заседания экспертного комитета при участии психолога и двух переводчиков, занятых на первом этапе прямого перевода, после чего выполнен ее обратный перевод на английский язык переводчиком — носителем английского языка, обладающим знаниями в области медицины и имеющим высокий уровень знания русского языка. На следующем этапе была осуществлена гармонизация переводов — экспертным комитетом с участием психолога и переводчика, выполнявшего обратный перевод, проведено обсуждение принципиальных расхождений, выявленных между оригинальной версией и обратным переводом, и утверждена тест-версия опросника. На основании экспертных оценок получены высокие показатели содержательной валидности русской версии PHPQoL — понятность, легкость и удобство оценки (для каждого вопроса и для инструмента в целом) и информативность инструмента в целом.

Для тестирования PHPQoL в процедуру интервьюирования были включены 5 пациентов — женщины в возрасте от 39 лет до 61 года, страдающие ПГПТ. В процессе интервьюирования все пациенты отметили ясность изложения и понятность смысла вопросов тест-версии на русском языке, положительное впечатление о предложенном способе оценки проблем, связанных с качеством жизни при данном заболевании, а также соответствие вопросов опросника актуальным проблемам у больных при ПГПТ. В среднем заполнение опросника пациентом составило 4 мин. На основании результатов тестирования PHPQoL при участии пациентов определены три показателя внешней валидности — понятность, легкость и удобство оценки (для каждого симптома и для инструмента в целом), а также полнота оценки (для инструмента в целом). Каждый показатель выражен в баллах от 0 до 1; чем выше показатель, тем лучше внешняя валидность инструмента по данной категории. В целом для русской версии PHPQoL получены высокие показатели внешней валидности: понятность — 1,0 балла, легкость и удобство оценки — 0,86 балла, полнота оценки — 0,9 балла. Общий (средний) показатель внешней валидности составил 0,87 балла. Выявленные в ходе тестирования комментарии пациентов по некоторым вопросам в целом являлись незначительными и были обусловлены, скорее всего, индивидуальными особенностями пациентов и внешними факторами, а не погрешностями перевода.

Таким образом, по результатам тестирования PHPQoL при участии пациентов, а также в ходе экспертной оценки клиницистами была подтверждена приемлемость русской версии PHPQoL, ее соответствие этнолингвистической среде, высокие показатели содержательной и внешней валидности [18]. Образец адаптированной русской версии опросника PHPQoL представлен в Приложении 1.

## Апробация опросника PHPQoL с оценкой его психометрических свойств

В апробации русской версии опросника PHPQoL приняли участие 65 больных ПГПТ, средний возраст больных — 52,3±10,5 года, 97% составили женщины. В табл. 1 представлена характеристика выборки.

**Table:** Таблица 1. Характеристика выборки

Показатели	Значение
Общее количество больных, n (%)	65 (100%)
Возраст, среднее значение (стандартное отклонение), лет	52,3 (10,5)
Женщины, n (%)	63 (96,9)
ECOG*, n (%) 0 1 2	21 (32,3) 43 (66,2) 1 (1,5)
Форма заболевания, n (%) Малосимптомная	21 (32,3)
Манифестная	44 (67,7)
Гиперкальциемия, n (%) Легкая Умеренная Тяжелая	42 (64,6) 20 (30,8) 3 (4,6)
Артериальная гипертензия, n (%)	35 (53,8)
Хроническая сердечная недостаточность, n (%)	13 (20,0)
Сопутствующие заболевания**, n (%)	41 (63,1)

Примечания: *ECOG — общесоматический статус; **среди сопутствующих заболеваний — патология желудочно-кишечного тракта, n=23 (хронический гастрит, хронический гастродуоденит, гастроэзофагеальная рефлюксная болезнь, язвенная болезнь желудка и двенадцатиперстной кишки, холецистит); эндокринная патология, n=11 (карцинома щитовидной железы, эутиреоз, сахарный диабет, аденома гипофиза, ожирение); почечная патология, n=4; патология суставов, n=2; хроническая обструктивная болезнь легких, n=1.

Среднее значение ионизированного кальция составило 1,5±0,15 ммоль/л, среднее значение паратиреоидного гормона (ПТГ) — 102,1±124,9 пг/мл.

Рассмотрим результаты апробации русской версии опросника PHPQoL в фокусной группе больных ПГПТ. В целом опросник был понятен и не вызывал трудностей в заполнении у подавляющего большинства пациентов. Опросники были заполнены полностью в подавляющем большинстве случаев: пропущенных данных на всем массиве — 0,7%; 86,2% больных ответили на все вопросы опросника, что сопоставимо с данными, полученными при использовании оригинальной версии опросника (88%; S. Webb и соавт., 2016). Полученные результаты свидетельствуют о высоком качестве данных и хорошей заполняемости опросника.

Ниже представлены результаты оценки психометрических свойств русской версии опросника PHPQoL.

## Конструктная валидность

Для анализа структуры русской версии опросника PHPQoL был проведен разведочный факторный анализ с использованием метода главных компонент и способа вращения варимакс по критерию каменистой осыпи. Анализ структуры опросника проведен с использованием 16 пунктов, отражающих симптомы/проблемы, связанные с ПГПТ, и влияющих на качество жизни пациентов при данном заболевании. В ходе факторного анализа извлечены 5 факторов (фактор I — пункты 14 и 16; фактор II — пункты 3, 5 и 6, фактор III — пункт 15, фактор IV — пункты 12 и 13, фактор 5 — пункты 1, 2 и 7–11). Величины факторных нагрузок для пунктов, относящихся к одному фактору, соответствовали значениям в диапазоне от 0,58 до 0,863. Пункт 15 («Я переживал(а) не только из-за болезни, но и из-за ее возможных осложнений») не относится ни к одному из перечисленных факторов и представляет собой самостоятельный вопрос с высоким значением факторной нагрузки (факторная нагрузка — 0,914). Пункт 4 («Я замечал(а), что задыхаюсь, если мне приходится идти быстро») группируется сразу двумя факторами — фактором IV и фактором V (факторные нагрузки — 0,453 и 0,380). Четыре фактора и один отдельный пункт описывают 73% дисперсии. При поочередном удалении одного из 3 пунктов, сгруппированных фактором II, а также одного из 3 пунктов, сгруппированных фактором IV, и одного из 7 пунктов, сгруппированных фактором V, отмечено уменьшение величины стандартизованного коэффициента α-Кронбаха для соответствующего фактора, что свидетельствует об устойчивой структуре инструмента.

Таким образом, показана устойчивая структура инструмента, свидетельствующая об удовлетворительной конструктной валидности русской версии опросника PHPQoL. Устойчивость структуры инструмента свидетельствует о корректном проведении языковой и культурной адаптации на предыдущем этапе и является основой для возможности проведения корректной оценки симптомов/проблем, влияющих на качество жизни, у больных ПГПТ.

## Надежность

При анализе надежности методом оценки внутреннего постоянства для 16 вопросов, связанных с симптомами/проблемами при ПГПТ, получено значение коэффициента α-Кронбаха, равное 0,87. Полученная величина коэффициента α-Кронбаха свидетельствует о высоком внутреннем постоянстве русской версии PHPQoL и даже превышает таковой показатель, полученный при валидации оригинальной версии опросника (0,80) [14]. В табл. 2 представлены значения коэффициента α-Кронбаха при поочередном удалении пунктов опросника. Можно утверждать, что инструмент позволяет осуществлять достаточно точную оценку симптомов/проблем, оказывающих влияние на качество жизни больных ПГПТ.

**Table:** Таблица 2. Значения коэффициента α-Кронбаха при удалении пунктов опросника PHPQoL

	Пункты опросника	Стандартизованный коэффициент α-Кронбаха: 0,88
1	Я испытывал(а) сонливость после того, как просыпался(ась) утром, и мне было трудно начинать день	0,86
2	Я ощущал(а) слабость	0,86
3	Я замечал(а), что мне тяжело долго ходить	0,87
4	Я замечал(а), что задыхаюсь, если мне приходится идти быстро	0,87
5	Я испытывал(а) боль в спине	0,87
6	У меня болели кости и суставы	0,87
7	Я замечал(а), что мне тяжело выполнять свои ежедневные дела	0,86
8	Я ограничивал(а) свой досуг	0,86
9	Я ограничивал(а) свои домашние обязанности	0,86
10	Я чувствовал(а) раздражительность	0,87
11	Я бывал(а) в плохом настроении/депрессии	0,86
12	Из-за болезни я страдал(а) бессонницей	0,87
13	Я просыпался(ась) ночью	0,87
14	Я замечал(а), что мне бывает трудно сконцентрироваться	0,86
15	Я переживал(а) не только из-за болезни, но и из-за ее возможных осложнений	0,88
16	Я замечал(а), что мне бывает труднее сконцентрироваться на работе, чем раньше	0,86

## Критериальная валидность

Для оценки критериальной валидности провели анализ связи суммарного балла по опроснику с количеством испытываемых пациентами симптомов и проблем вследствие заболевания. Среди наиболее частых симптомов у пациентов исследуемой группы были чувство постоянной усталости (61%), слабость (54%), боли в суставах (53%) и нарушение памяти (52%). При анализе корреляций между суммарным баллом по PHPQoL и количеством симптомов у больных ПГПТ выявлена статистически значимая отрицательная слабая связь (r=-0,46; p<0,001). Чем больше симптомов и проблем, связанных с заболеванием, испытывает пациент, тем хуже его показатели качества жизни. Полученный результат отражает хорошую критериальную валидность русской версии опросника PHPQoL.

## Дискриминантная валидность

В основе анализа дискриминантной валидности русской версии опросника PHPQoL была оценка чувствительности инструмента к клинически обоснованным различиям между группами больных ПГПТ. В ходе анализа методом «известных групп» выполняли сравнение суммарного балла по PHPQoL в группах больных с малосимптомной и манифестной формами заболевания: суммарный балл качества жизни по опроснику PHPQoL у больных с малосимптомной формой заболевания выше, чем у больных с манифестной формой заболевания, — 64,7 против 47,4 (парный тест Стьюдента, p=0,01). Таким образом, продемонстрирована способность инструмента определять разный уровень качества жизни у больных в зависимости от наличия или отсутствия проявлений болезни. Эти данные характеризуют удовлетворительную дискриминантную валидность русской версии опросника.

## Конвергентная валидность

В процессе корреляционного анализа выявлены статистически значимые положительные корреляции между суммарным показателем по PHPQoL и всеми показателями по шкалам опросника SF-36:

Таким образом, можно утверждать, что в результате апробации показана удовлетворительная конвергентная валидность русской версии опросника PHPQoL.

## Чувствительность

Анализ чувствительности русской версии опросника PHPQoL к изменениям во времени проводили на основании оценки изменений суммарного показателя по PHPQoL в группе пациентов, которые заполнили опросник до и через 3 мес после операции (n=23). Через 3 мес после ПТЭ выявлено статистически значимое увеличение суммарного показателя качества жизни — 46,7 против 55,7 (p=0,043), что свидетельствует о значимом разрешении симптомов/проблем у пациентов данной группы после хирургического лечения ПГПТ.

Также проанализировали количество пациентов, у которых выявлено увеличение суммарного показателя по опроснику PHPQoL на 9 и более баллов, что считается проявлением клинически значимого улучшения качества жизни у больных ПГПТ в процессе лечения [15]. Клинически значимое улучшение качества жизни зарегистрировано у 44% прооперированных больных.

Чувствительность инструмента к изменениям в состоянии больных во времени, в том числе в процессе лечения, является важным психометрическим свойством опросника, во многом определяющим возможности его применения в клинической практике и исследованиях для оценки динамики качества жизни у больных, а также при оценке эффекта лечения. В целом на основании полученных результатов можно сделать вывод о том, что русская версия опросника PHPQoL чувствительна к изменениям в состоянии пациентов с ПГПТ, обусловленным эффектом проведенного хирургического лечения, и может использоваться в практических целях как дополнительный критерий эффективности лечения.

На заключительном этапе апробации опросника PHPQoL проведен анализ показателей качества жизни у больных ПГПТ до и через 3 мес после оперативного вмешательства. Суммарный показатель качества жизни по опроснику PHPQoL больных до операции составил 46,7±17,4 балла (диапазон от 0 до 100 баллов). При этом показатель физического компонента качества жизни — 45,5±19,7 балла; психического компонента качества жизни — 48,2±16,8 балла. Через 3 мес после операции имело место значимое улучшение качества жизни больных — суммарный показатель качества жизни увеличился до 55,7±14,8 балла. При этом произошло улучшение как по физическому, так и психологическому компонентам качества жизни. Показатель физического компонента качества жизни увеличился до 55,2±19,2 балла, а психического — до 56,6±15,7 балла.

На рисунке 2 представлено распределение больных согласно суммарному показателю качества жизни до и после операции. Как видно из рисунка, до операции у 12% больных суммарный показатель находился в диапазоне 0–25 баллов, у 44% больных — 26–50 баллов, у 38% больных — 51–75 и у 6% больных — 76–100 баллов. После операции существенно снизилась доля больных с низкими баллами и увеличилась доля больных с высокими баллами качества жизни. Отметим, что у большинства больных (69%) суммарный показатель качества жизни через 3 мес после операции регистрировался в диапазоне 51–75 баллов.

**Figure:**
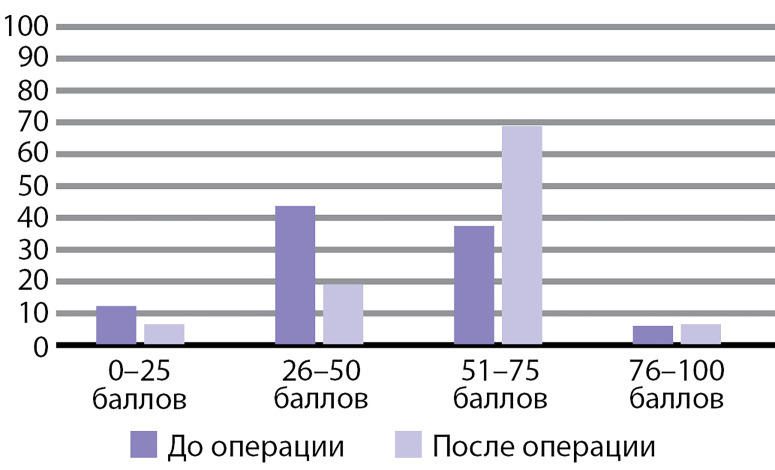
Рис. 2. Гистограмма распределения пациентов согласно величине суммарного показателя качества жизни по опроснику PHPQoL в группе больных первичным гиперпаратиреозом до операции и через 3 мес после операции.

Таким образом, применение опросника PHPQoL до и после оперативного вмешательства позволяет мониторировать изменения качества жизни больных ПГПТ и оценивать динамику физического и психологического функционирования после проведенного лечения.

## ОБСУЖДЕНИЕ

Результаты целого ряда исследований свидетельствуют о том, что хирургическое лечение приводит к улучшению качества жизни пациентов с ПГПТ [9–11][19][20]. В работах Ryhänen E. и соавт. [19] и Ejlsmark‐Svensson H. и соавт. [20] продемонстрировано, что оценка качества жизни пациентов с ПГПТ с использованием стандартизированных опросников до и в разные сроки после ПТЭ позволяет оценить эффективность оперативного вмешательства.

В результате исследования проведены валидация и апробация русской версии опросника PHPQoL для оценки симптомов/проблем, влияющих на качество жизни у больных ПГПТ, и выполнено тестирование психометрических свойств опросника. В ходе апробации инструмента показана хорошая его заполняемость пациентами, требующая немного времени, а также понятность и легкость при выборе пациентами ответов на вопросы.

Методы, выбранные для валидации русской версии опросника PHPQoL, основаны на подходах, использованных при проверке психометрических свойств оригинальной версии опросника [14][15], и ориентированы на определение информативности инструмента при ведении больных ПГПТ в клинической практике. Ключевыми психометрическими характеристиками, которые проверяли в ходе тестирования русской версии опросника PHPQoL для оценки пригодности его применения в клинической практике, являлись надежность, т.е. способность инструмента выполнять точную оценку симптомов/проблем у больного ПГПТ, а также способность инструмента отражать клинические различия в состоянии больных (дискриминантная и критериальная валидность) и чувствительность к изменению в состоянии больных во времени.

Важным результатом исследования является продемонстрированная устойчивость структуры инструмента, свидетельствующая об удовлетворительной конструктной валидности русской версии и в существенной степени определяющая приемлемость ее использования для оценки симптомов/проблем у больных ПГПТ. Результаты оценки внутреннего постоянства инструмента указывают на надежность русской версии опросника PHPQoL. Полученная величина коэффициента α-Кронбаха (0,87) сопоставима с данными, полученными для оригинальной версии опросника [14], и характеризует высокое внутреннее постоянство его русской версии, т.е. высокую точность получаемых результатов.

В ходе исследования продемонстрирована возможность определять различия показателей симптомов/проблем ПГПТ между пациентами в зависимости от клинических признаков, а также зависимость суммарного балла по опроснику от количества испытываемых пациентами симптомов/проблем, связанных с заболеванием, что является очень важным свойством опросника и свидетельствует о достоверности и информативности данных, которые можно получить при его использовании у пациентов с разной формой заболевания и при разной выраженности его проявлений. Важно отметить, что схожие результаты были продемонстрированы при валидации оригинальной версии опросника PHPQoL [15]. Актуальными и обоснованными в контексте анализа конвергентной валидности являются полученные нами данные корреляционного анализа между суммарным баллом по PHPQoL и показателями шкал опросника SF-36. По аналогии с результатами, показанными ранее для оригинальной версии инструмента, нами установлена связь между показателями симптомов/проблем по PHPQoL и показателями шкал качества жизни опросника SF-36: чем выше выраженность симптомов/проблем по суммарному баллу опросника, тем ниже показатели по шкалам опросника SF-36 [15].

Важным результатом валидации русской версии опросника PHPQoL является продемонстрированная способность инструмента определять изменения показателей симптомов/проблем по PHPQoL в процессе лечения. По аналогии с результатами, полученными при апробации оригинальной версии опросника у пациентов с ПГПТ после хирургического лечения, через 3 мес после операции наблюдали значительное улучшение показателей качества жизни [15]. Интересно, что улучшение качества жизни больных наблюдали уже через 3 мес после ПТЭ — эти данные дополняют результаты, полученные в исследовании Ejlsmark‐Svensson H. и соавт. [20], согласно которым качество жизни больных значимо улучшалось через 12 мес после операции. Таким образом, полученные данные открывают перспективу применения русской версии PHPQoL для оценки ответа на лечение у больных ПГПТ, в том числе в отдаленный период после удаления доброкачественного новообразования.

В ходе исследования продемонстрирован положительный эффект хирургического лечения на качество жизни больных ПГПТ. Увеличение суммарного показателя качества жизни по опроснику PHPQoL свидетельствует о разрешении симптомов/проблем у пациентов данной группы после хирургического лечения; почти у половины больных зарегистрировано существенное улучшение качества жизни после операции по сравнению с исходными показателями.

Полученные результаты находятся в соответствии с данными валидационного исследования Webb S.M. и соавт., согласно которому показатели качества жизни пациентов с ПГПТ после ПТЭ улучшаются уже через 3 мес, и данные положительные изменения, согласно данным опросника PHPQoL, сохраняются на протяжении последующих 9 мес [15].

Таким образом, все результаты, полученные в ходе апробации и валидации, позволяют утверждать, что валидированная в соответствии с международными стандартами и апробированная в российской популяции больных ПГПТ русская версия опросника PHPQoL является надежным, валидным и чувствительным инструментом для оценки симптомов/проблем у пациентов с ПГПТ и может использоваться в клинической практике и научных исследованиях в отечественной эндокринологии.

## Направления дальнейших исследований

Перечень задач, которые представляют сегодня большую практическую значимость при ведении больных ПГПТ и решение которых становится возможным при использовании опросника PHPQoL, включает, в том числе, анализ факторов, связанных с качеством жизни у больных ПГПТ, а также изучение закономерностей изменения качества жизни у пациентов в отдаленный период после операции в зависимости от проявлений заболевания и нарушения разных аспектов качества жизни до операции.

## ЗАКЛЮЧЕНИЕ

С применением многоэтапной процедуры перевода, культурной и языковой адаптации и валидации была разработана русская версия инструмента для оценки качества жизни у больных ПГПТ — PHPQoL. Соблюдение принципов культурной и языковой адаптации позволило максимально приблизить концепцию данного инструмента к культурным и языковым традициям и особенностям популяции больных данной категории в нашей стране. В результате валидации удалось продемонстрировать, что русская версия PHPQoL является надежным, валидным и чувствительным инструментом для оценки симптомов/проблем у пациентов с ПГПТ.

Валидированная русская версия опросника PHPQoL может применяться в российской популяции больных ПГПТ как в научных исследованиях, так и клинической практике. Имеются перспективы ее использования при мониторинге больных с целью оценки эффекта терапии, в том числе после хирургического лечения.

Хирургическое лечение значительно улучшает качество жизни пациентов с ПГПТ. Применение опросника PHPQoL в клинической практике у больных ПГПТ до и после операции может способствовать реализации пациентоориентированного подхода при ведении больных ПГПТ и совершенствованию отечественной системы медицинской помощи этой категории пациентов.

## ПРИЛОЖЕНИЕ 1

**Table:** Образец адаптированной русской версии опросника качества жизни при первичном гиперпаратиреозе (PHPQoL)

		0 —всегда	1 —очень часто	2 — время от времени	3 —очень редко	4 — никогда
1	Я испытывал(а) сонливость после того, как просыпался(ась) утром, и мне было трудно начинать день					
2	Я ощущал(а) слабость					
3	Я замечал(а), что мне тяжело долго ходить					
4	Я замечал(а), что задыхаюсь, если мне приходится идти быстро					
5	Я испытывал(а) боль в спине					
6	У меня болели кости и суставы					
7	Я замечал(а), что мне тяжело выполнять свои ежедневные дела					
8	Я ограничивал(а) свой досуг					
9	Я ограничивал(а) свои домашние обязанности					
10	Я чувствовал(а) раздражительность					
11	Я бывал(а) в плохом настроении/депрессии					
12	Из-за болезни я страдал(а) бессонницей					
13	Я просыпался(ась) ночью					
14	Я замечал(а), что мне бывает трудно сконцентрироваться					
15	Я переживал(а) не только из-за болезни, но и из-за ее возможных осложнений					
16	Я замечал(а), что мне бывает труднее сконцентрироваться на работе, чем раньше				
